# Duration of agricultural pesticide exposure application and Parkinson’s disease in California’s central valley

**DOI:** 10.1088/2752-5309/ae4645

**Published:** 2026-03-03

**Authors:** Yufan Gong, Kimberly C Paul, Irish Pearl Cambronero Del Rosario, Keren Zhang, Myles G Cockburn, Laura K Thompson, Adrienne M Keener, Jeff M Bronstein, Beate R Ritz

**Affiliations:** 1 Department of Epidemiology, Fielding School of Public Health, University of California, Los Angeles, CA 90095, United States of America; 2 Department of Neurology, David Geffen School of Medicine, University of California, Los Angeles, CA 90095, United States of America; 3 Department of Population and Public Health Sciences, Keck School of Medicine, University of Southern California, Los Angeles, CA 90033, United States of America; 4 Department of Environmental Health Science, Fielding School of Public Health, University of California, Los Angeles, CA 90095, United States of America

**Keywords:** pesticide, duration, lagging, Parkinson’s disease

## Abstract

While numerous pesticides have been linked to Parkinson’s disease (PD), few studies have evaluated the long-term effects of ambient exposure to the wide range of pesticides used in commercial agriculture across multiple decades and exposure windows. We examined the contribution of exposure duration and timing to PD risk in 829 PD patients and 824 controls enrolled in California’s Central Valley. Using a validated geospatial model integrating California pesticide use reporting and land-use data, we quantified proximity-based ambient pesticide applications at residential and workplace addresses for 287 pesticides applied within 500 m of at least 25 participants’ locations between 1974 and 10 years prior to the index year (diagnosis for cases, enrollment for controls, 1998–2016). The exposure duration was calculated as the proportion of eligible years with any nearby application. Unconditional logistic regression models (false discovery rate (FDR)-corrected) estimated exposure effects for each pesticide on PD risk, and constrained distributed lag models evaluated associations across time windows before diagnosis. Longer exposure duration to 56 pesticides was associated with increased PD risk (FDR < 0.05), with odds ratios ranging from 1.10 to 1.25 per standard deviation increase in exposure. Among these, 34 overlapped with pesticides previously identified using an intensity-based metric. Endothall showed the strongest association (OR = 1.27, 95% CI: 1.16–1.39). Associations were generally stronger for exposures occurring 11–20 or 21–30 years prior to diagnosis. These findings indicate that prolonged exposure duration to specific pesticides is relevant to PD risk and suggest latency periods of two to three decades for several agents.

## 1. Introduction

Parkinson’s disease (PD) is one of the most prevalent neurodegenerative disorders worldwide and is currently among the fastest-growing neurologic conditions in aging populations [1–3]. Heritability estimates suggest that genetic variants account for approximately 10% to 30% of the variation in PD risk, leaving a substantial portion for environmental influences in addition to aging and stochastic processes [4–6]. The evidence for a role of environmental contributors to PD is growing and especially compelling for pesticide exposures [7–9]. A 2022 meta-analysis, which included 8 cohort and 24 case-control studies, reported that pesticide exposure in general was associated with a 1.34- to 1.56-fold increased risk of PD, depending on study design and exposure assessment methods [10].

Despite the repeated findings of associations, substantial research gaps remain. First, many studies relied on crude exposure metrics (self-reported, ever vs never, high vs low, using broad occupational or chemical categories, etc.), which are prone to exposure misclassification and recall bias and do not capture the complexity of exposure scenarios in terms of intensity, duration, or timing of exposure [11–13]. Additionally, few studies have addressed individual pesticides and those that did mostly focused on a few specific agents or relied on broad groups of pesticides in which members may differ greatly in terms of neurotoxicity [14]. We previously conducted and published an untargeted pesticide-wide association study (PWAS), an agnostic scan across many pesticides, where hundreds of pesticide active ingredients were evaluated using a record-based proximity model and an exposure intensity metric (average annual pounds applied within 500 m of participants’ residences and workplaces) [9]. The PWAS identified 53 pesticides that were formally statistically associated with PD (FDR < 0.05). In addition, correlation analysis revealed several distinct co-exposure patterns in this agricultural region. Follow-up experimental studies identified 10 toxins which were directly toxic to dopaminergic neurons. However, we did not assess the role of exposure duration to determine whether long-term exposures are potentially as important as higher exposures in terms of intensity.

Furthermore, there is also a lack of understanding whether there are exposure periods that are most relevant in PD, i.e. sensitive windows for chronic exposures. Studies often rely on cumulative exposure metrics and do not address whether exposures and their effects change over time. This is particularly important given the known prolonged prodromal period of PD, which often spans decades between the initial onset of non-motor symptoms and diagnosis [15].

Our study leverages a large case-control study with participants residing in California’s Central Valley, where detailed agricultural pesticide application records are available over more than four decades. Together with land use information, we previously employed these data in the study region in a geographic information system (GIS)-based exposure model and evaluated PD risk for pesticide application intensity (yearly pounds of agricultural pesticides applied). Here we quantify these proximity-based ambient exposures in terms of duration (number of years with exposure), providing information beyond our previous analyses [9]. Additionally, for the first time, we explore sensitive windows of exposure in the decades prior to PD onset to improve our understanding of how pesticides may impact PD risk.

## 2. Methods

### 2.1. Study population

The Parkinson’s Environmental and Gene (PEG) Study, a population-based case-control study, is comprised of 1,653 participants (829 PD patients and 824 controls) enrolled in two separate study waves (PEG1: 2000–2007, PEG2: 2010–2016). The study was designed to investigate the influence of pesticides on PD risk among people residing in three agricultural counties of California’s Central Valley (Kern, Tulare, and Fresno).

Details on recruitment and enrollment have been provided elsewhere [16–19]. To be eligible, PD patients had to: (1) live in the tri-county area at time of contact; (2) have lived in CA at least 5 years prior to PD diagnosis; and (3) be in early stages of PD (i.e. first diagnosed ⩽3 years prior to recruitment in PEG1, and ⩽5 years in PEG2). Eligible PD patients who agreed to participate were examined at least once but most several times by movement disorder specialists from University of California, Los Angeles (UCLA) and determined to have clinically possible or probable idiopathic PD. In this study, a total of 357 PD cases in PEG1 and 472 in PEG2 were included and provided with all information needed.

Population controls were identified first from Medicare lists (2001) and later from residential tax assessor records due to the implementation of the Health Insurance Portability and Accountability Act (HIPAA). To be eligible, controls were required to be (1) at least 35 years of age; (2) live in the same tri-county area; (3) have lived in CA for at least 5 years prior to enrollment; and (4) not having received a Parkinsonism diagnosis. In the current study, 400 controls from PEG1 and 424 from PEG2 were included and provided with all necessary information.

More information regarding screening and inclusion criteria can be found in the supplementary material.

### 2.2. Pesticide exposure estimation

In 1972, California enacted legislation which required mandatory reporting of all pesticide applications by licensed applicators and all applications of restricted-use pesticides in production agriculture. Since 1974, the information on pesticide use was collected by the California Agricultural Pesticide Use Reporting (CA-PUR) system, which records the active ingredient, registration number, date and location of applications, along with the poundage applied and other information. In 1990, the mandatory reporting was extended for all pesticide usage [20].

Based on the CA-PUR, we estimated ambient pesticide exposure using a GIS-based computer model we developed and validated previously [21–23]. In brief, the estimation process includes the following steps: (1) record each participant’s lifelong residential and occupational address histories by interview; (2) geocode the eligible addresses to latitude and longitude; (3) link latitude and longitude to the CA-PUR database combined with land-use survey data to generate annual measures of pounds per acre of each active ingredient applied within a 500 m radius buffer around each location. This 500 m buffer distance was selected based on prior methodological work, demonstrating that this buffer distance captures relevant off-site exposures from pesticide applications while limiting exposure misclassification arising from spatial uncertainty in application location and land-use data [23]. Out of the 1355 pesticides used between 1974 and 2017, 722 were applied within a 500 m radius of at least one PEG participant’s home or workplace address. Among them, a total of 287 pesticides qualified for analysis, as we required that at least 25 people had records of any exposure at either residential or workplace addresses. Detailed categorization (including toxicity groups, usage type and chemical classes) of pesticides has already been established in our prior work using the same GIS-based exposure model [9]. Paraquat dichloride was not included among these 287 pesticides for the following analyses because we have recently reported on how duration and intensity of paraquat exposure affect PD risk [24].

In this study, ‘ambient pesticide exposure’ refers to a proximity-based environmental exposure metric derived from recorded pesticide applications near participants’ residences and workplaces. This measure does not assume or distinguish specific exposure pathways or routes. Rather, it reflects overall exposure potential from nearby agricultural pesticide use that may result in pesticide drift and inhalation or ingestion of air or dust that contains these pesticides.

PD develops gradually over many years prior to clinical diagnosis. Substantial neurodegenerative processes are believed to occur during this preclinical phase, and non-motor symptoms may precede diagnosis by a decade or longer [1]. Consequently, pesticide exposures occurring shortly before diagnosis are less likely to contribute to disease initiation and may instead reflect exposures that occur after relevant pathogenic processes have already begun. In addition, since participants entered the study over a lenghty enrollment period (2000–2016), were only required to have resided in California for a minimum of five years prior to enrollment, and statewide pesticide use reporting data were only available beginning in 1974, the total number of years during which pesticide exposure could be observed varied substantially across individuals.

To ensure comparability of exposure duration across participants with heterogeneous observable length of time at risk, we included a 10 year lag to account for the prodromal period of PD and defined exposure duration as the proportion of eligible years during which a participant lived or worked within 500 m of pesticide application, rather than as the absolute number of exposed years. By using a proportion-based metric, we standardized exposure to the individual’s observed time at risk during which proximity to applications was known, allowing us to capture the percentage of time a participant lived or worked near pesticide applications according to the presumed exposure window.

Specifically, for each pesticide, we calculated the number of years in which any application occurred within a 500 m buffer of a participant’s residential or occupational address between 1974 and 10 years prior to the index year, with the index year being the year of diagnosis for the patients and enrollment for controls (median: 2007, IQR: 2004–2009). This value was then divided by the total number of years the participant had an eligible address during this period:

\begin{equation*}{\mathrm{Duratio}}{{\mathrm{n}}_i} = { }\frac{{{N_i}}}{{{T_i}}}\end{equation*}

where $i \in \left( {1:1,653} \right)$ (i.e. $i{\mathrm{th}}$ participant in the study sample), $N$ represents the number of years with any application of the respective pesticide in the 500 m buffer zone around an address and $T$ stands for the number of years that a participant had an eligible address in the exposure period.

Thus, the duration measure ranges from 0 to 1, where 1 means the participant lived or worked near (<500 m) application of the pesticide every single year of the exposure period and 0 means no exposure in any eligible year. Then we created a *Z* score for each pesticide (i.e., centered the duration mean to 0 and scaled the duration estimate to its standard deviation (SD)) such that each estimate can be interpreted as representing a per 1 SD increase in duration percentage from the mean.

For sensitivity analysis, we first additionally constructed another weighted duration metric to better account for differences in the length of individual exposure windows. Specifically, we treated the proportion-based duration measure (i.e. ${N_i}/{T_i}$) as a weight and multiplied it by the log-transformed number of years exposed, offset by 1 (i.e. log (${N_i} + 1$)). This approach reduces the influence of short observation windows, as with the other measure even a single year of exposure would yield a proportion-based duration value of 1 if there was only one year in the exposure period left after applying a 10 year lag.

Then, we excluded participants with short exposure histories to evaluate the potential influence of limited observable exposure windows on the proportion-based duration metric. Specifically, participants with fewer than 5 or 10 observable exposure years in PEG1 and in PEG2 were excluded from these analyses. This resulted in the exclusion of 53 and 156 participants, respectively. Meta-analyses were then repeated using the same modeling framework as in the primary analyses to assess the robustness of associations to these exclusions.

To explore lagged effects for each pesticide, we created exposure windows by subtracting the year of exposure from the index year for each participant and categorizing them into 4 intervals of 10 year length (i.e. exposure window years: 1–10, 11–20, 21–30, and 31–40 years prior to index). For pesticide exposure within the windows, we applied the proportion-based duration measure and additionally calculated the yearly average pounds of each active ingredient applied per acre during the valid exposure period. Furthermore, while cumulative exposure duration incorporated both residential and workplace addresses to better characterize long-term ambient exposure burden, we focused on residential-based proximity to pesticide applications only for this analysis. This is because PD patients often reduce or stop working prior to diagnosis, such that the exposure window is systematically shorter for patients than controls [9]. Including workplace exposure in lagged analysis could therefore distort temporal exposure patterns by introducing differential exposure window lengths for cases and controls. Residential address histories, on the other hand, were comparable in presumed exposure window length for participants.

### 2.3. Statistical analysis

We first estimated the cumulative effect for each of the 287 selected pesticides on PD versus no PD. First, we used univariate, unconditional logistic regression models, in which we adjusted for age at diagnosis/interview, gender, ethnicity (non-Hispanic White, Hispanic, Other), education (in years), smoking status (former smoker, current smoker, and non-smoker), study wave (PEG1 & PEG2), and index year (year of diagnosis for PD patients/year of interview for non-disease controls). Specifically, we fit the following logistic regression models:

\begin{equation*}{\mathrm{logit}}\left( {P = 1{\mathrm{|}}{X_m},{ }Z} \right) = {\alpha _0} + {\alpha _m}{X_m} + {\alpha _z}Z\end{equation*}

where ${X_m}$ is the $m{\mathrm{th}}$ [*m* $ \in \left( {1:287} \right)$] vector of pesticide exposure for each study subject, and *Z* stands for the matrix of potential confounders.

Log odds and SDs were determined separately for each exposure location (residential and occupational); then the estimates for each pesticide were combined in a fixed-effect meta-analysis. We took into consideration multiple comparisons by applying the false discovery rate (FDR) correction.

To compare the results from the duration-based exposure assessment with our previously reported findings using an intensity-based exposure measure, we calculated the ratio of odds ratios (RORs) and conducted a paired *t*-test to determine whether ORs from the duration-based measure differed from their intensity equivalents. Additionally, we conducted Bland–Altman analyses comparing odds ratio (OR) estimates derived from the duration-based exposure measure used in the current study with those derived from the previously published intensity-based exposure measure. Specifically, for each pesticide, the RORs from the two exposure metrics were plotted against their geometric mean, allowing assessment of systematic differences and overall agreement across the range of effect estimates. Limits of agreement were calculated to quantify variability between the two approaches.

Then we conducted lagged exposure analyses to account for the long latency of PD and to evaluate whether exposure during specific time windows prior to diagnosis was differentially associated with PD risk. Specifically, we applied constrained distributed lag models (DLMs) to assess time-varying associations of pesticides and PD onset, using cross-basis functions to describe the two-dimensional feature for exposure and lag effects employing the *dlnm* package [25]. We chose a simple linear function to model the exposure-response relationship and a natural cubic spline with knots at 2 and 3 (i.e., for the 11–20 and the 21–30 years windows) to model the lag-response relationship. The intercept for the lag-response function was set to FALSE to specify a null effect for the first lag period (i.e., ignoring exposures within a 10 year prodromal period as discussed above).

In addition, for comparisons to evaluate the influence of the model on our results, we also explored three additional modeling approaches for time-window analyses: (1) univariate logistic models for each exposure window with and without adjusting for exposure in the other windows; (2) penalized ridge regression models with 20-fold cross-validation to reduce the variance introduced by correlated exposures across exposure windows; (3) treed-distributed regression models suggested by Mork and Wilson [26] that do not require a smoothness assumption for the spline functions in the constrained DLMs and allows us to identify the most relevant exposure windows more accurately. These analyses were designed to characterize temporal patterns of association that may be consistent with potential disease latency periods by allowing exposure-disease associations to vary across predefined pre-diagnostic intervals.

All data analyses were performed in R 4.3.3 (R Foundation for Statistical Computing, Vienna, Austria).

## 3. Results

### 3.1. Demographic characteristics

As shown in table 1, the mean age at diagnosis for PD patients was 67.7 years ($ \pm $ 10.6) and for controls 65.9 ($ \pm $ 11.6) years at interview. Patients and controls were generally comparable in terms of race/ethnicity, county of residence, and education, but there were more men and non-smokers among the patients.

**Table 1.** Demographic characteristics of PEG participants (*N* = 1653).

	PD status	
Characteristics^a^	Without PD	With PD
	*N* = 824	*N* = 829
Age	65.9 (11.6)	67.7 (10.6)
Race/ethnicity		
White (not Hispanic)	569 (69.1%)	630 (76.0%)
Hispanic (any race)	155 (18.8%)	137 (16.5%)
Other	100 (12.1%)	62 (7.5%)
Study		
PEG1	400 (48.5%)	357 (43.1%)
PEG2	424 (51.5%)	472 (56.9%)
Sex		
Male	383 (46.5%)	524 (63.2%)
Female	441 (53.5%)	305 (36.8%)
School years	14 (4.0)	13.6 (4.4)
Smoker		
Non-smoker	397 (48.2%)	450 (54.3%)
Former smoker	331 (40.2%)	346 (41.7%)
Current smoker	96 (11.6%)	33 (4.0%)
County		
Kern	330 (40.0%)	318 (38.4%)
Fresno	348 (42.2%)	320 (38.6%)
Tulare	146 (17.7%)	191 (23.0%)

a Mean (SD); *n* (%).

### 3.2. Cumulative association between pesticides and PD in logistic regression models

Of the 287 pesticides examined, 56 were positively associated with PD based on the duration-based exposure measure at FDR < 0.05 in the fixed-effect meta-analysis. Table 2 presents the regression results of these individual PD-related pesticides along with their chemical classes. These associations corresponded to increases in the odds of PD between 10% and 25% per SD increase from the mean, based on exposure duration between 1974 and the decade preceding the index year. Among the 56 pesticides, organophosphates (OPs) (*n* = 14, including phorate, malathion, monocrotophos, chlorpyrifos, ethephon, mevinphos, phosmet, dimethoate, s,s,s-tributyl phosphorotrithioate, oxydemeton-methyl, parathion, methidathion, ethion, and diazinon) and petroleum derivatives (*n* = 6, including xylene range aromatic solvent, petroleum hydrocarbons, petroleum distillates (aromatic and refined), xylene, and mineral oil) appeared more frequently than other pesticide categories. The largest estimated effect size was observed for endothall (herbicide/defoliant, OR = 1.25 per SD, 95% CI = 1.15–1.35, FDR = 2.96 × 10^−05^), followed by pyriproxyfen (insect growth regulator, OR = 1.24 per SD, 95% CI = 1.14–1.35, FDR = 1.99 × 10^−04^), phorate (insecticide, OR = 1.21 per SD, 95% CI = 1.12–1.31, FDR = 2.73 × 10^−04^), sodium chlorate (herbicide/defoliant, OR = 1.20 per SD, 95% CI = 1.11–1.29, FDR = 2.99 × 10^−04^) and dicofol (insecticide, OR = 1.19 per SD, 95% CI = 1.10–1.28, FDR = 5.94 × 10^−04^). Model estimates stratified by exposure location for pesticides with FDR < 0.05 can be found in supplemental table 2.

**Table 2.** Estimated associations between pesticide and PD using duration.

Chemical name	Chemical class	OR	lowCL	upCL	FDR
Endothall, Mono (Diethyl Alkylamine)	Unclassified	1.25	1.15	1.35	2.96 × 10^−05^
Pyriproxyfen	Juvenile hormone mimic	1.24	1.14	1.35	1.99 × 10^−04^
Phorate	Organophosphorus	1.21	1.12	1.31	2.73 × 10^−04^
Sodium Chlorate	Inorganic	1.20	1.11	1.29	2.99 × 10^−04^
Dicofol	Organochlorine	1.19	1.10	1.28	5.94 × 10^−04^
Xylene Range Aromatic Solvent	Petroleum derivative	1.15	1.07	1.25	4.93 × 10^−03^
Petroleum Hydrocarbons	Petroleum derivative	1.16	1.07	1.26	4.93 × 10^−03^
Prometryn	Triazine	1.16	1.08	1.26	4.93 × 10^−03^
Carbaryl	N-Methyl Carbamate	1.15	1.07	1.24	4.93 × 10^−03^
Petroleum Distillates, Aromatic	Petroleum derivative	1.15	1.07	1.25	4.93 × 10^−03^
Carbofuran	N-Methyl Carbamate	1.17	1.08	1.26	4.93 × 10^−03^
Tebufenozide	Diacylhydrazine	1.17	1.08	1.27	4.93 × 10^−03^
Mcpa, Dimethylamine Salt	Chlorophenoxy acid	1.16	1.07	1.25	4.93 × 10^−03^
Mepiquat Chloride	Quaternary Ammonium	1.16	1.07	1.25	4.93 × 10^−03^
Propargite	Unclassified	1.15	1.07	1.24	4.95 × 10^−03^
Bromoxynil Octanoate	Hydroxybenzonitrile	1.15	1.07	1.24	5.48 × 10^−03^
Malathion	Organophosphorus	1.14	1.06	1.23	8.65 × 10^−03^
Monocrotophos	Organophosphorus	1.15	1.06	1.25	8.65 × 10^−03^
Chlorpyrifos	Organophosphorus	1.14	1.06	1.23	9.09 × 10^−03^
Ethephon	Organophosphorus	1.14	1.06	1.23	1.10 × 10^−02^
Diuron	Urea	1.14	1.05	1.22	1.15 × 10^−02^
Dicamba, Dimethylamine Salt, Other Related		1.14	1.05	1.23	1.20 × 10^−02^
Mevinphos	Organophosphorus	1.14	1.05	1.23	1.21 × 10^−02^
Mevinphos, Other Related		1.14	1.05	1.23	1.21 × 10^−02^
Trifluralin	2,6-Dinitroaniline	1.13	1.05	1.22	1.36 × 10^−02^
Pyridaben	Unclassified	1.14	1.05	1.24	1.36 × 10^−02^
Phosmet	Organophosphorus	1.13	1.05	1.22	1.44 × 10^−02^
Triadimefon	Azole	1.13	1.05	1.22	1.58 × 10^−02^
Dimethoate	Organophosphorus	1.13	1.05	1.22	1.58 × 10^−02^
S,S,S-Tributyl Phosphorotrithioate	Organophosphorus	1.14	1.05	1.23	1.58 × 10^−02^
Parathion, Other Related		1.13	1.05	1.22	1.58 × 10^−02^
Buprofezin	Unclassified	1.14	1.05	1.24	1.82 × 10^−02^
Aldicarb	N-Methyl Carbamate	1.12	1.04	1.21	1.93 × 10^−02^
Xylene	Petroleum derivative	1.12	1.04	1.21	2.48 × 10^−02^
Spinosad	Macrocyclic Lactone	1.12	1.04	1.21	2.48 × 10^−02^
Methomyl	N-Methyl Carbamate	1.12	1.04	1.21	2.53 × 10^−02^
Copper Sulfate (Pentahydrate)	Inorganic-Copper	1.13	1.04	1.23	2.53 × 10^−02^
Oxydemeton-Methyl	Organophosphorus	1.12	1.04	1.21	2.57 × 10^−02^
Phosphoric Acid	Inorganic	1.12	1.04	1.22	2.57 × 10^−02^
Phthalic Glycerol Alkyl	Unclassified	1.12	1.04	1.22	2.59 × 10^−02^
Parathion	Organophosphorus	1.12	1.04	1.21	2.61 × 10^−02^
Methidathion	Organophosphorus	1.11	1.03	1.20	2.98 × 10^−02^
2,4-D, Dimethylamine Salt	Chlorophenoxy acid	1.12	1.03	1.20	2.98 × 10^−02^
Petroleum Distillates, Refined	Petroleum derivative	1.15	1.05	1.28	2.98 × 10^−02^
Urea Dihydrogen Sulfate	Inorganic	1.12	1.04	1.22	3.08 × 10^−02^
Mineral Oil	Petroleum derivative	1.11	1.03	1.20	3.14 × 10^−02^
Fenpropathrin	Pyrethroid	1.11	1.03	1.20	3.41 × 10^−02^
1,2-Dichloropropane, 1,3-Dichloropropene	Halogenated organic	1.11	1.03	1.20	3.60 × 10^−02^
Msma	Organoarsenic	1.11	1.03	1.19	3.85 × 10^−02^
Dinoseb	Dinitrophenol derivative	1.11	1.03	1.20	4.11 × 10^−02^
Ethalfluralin	2,6-Dinitroaniline	1.13	1.03	1.23	4.11 × 10^−02^
Alpha-Octylphenyl-Omega-Hydroxypoly	Polyalkyloxy Compound	1.11	1.03	1.20	4.11 × 10^−02^
Dicamba, Dimethylamine Salt	Benzoic acid	1.11	1.03	1.19	4.18 × 10^−02^
Ethion	Organophosphorus	1.11	1.03	1.21	4.37 × 10^−02^
Diazinon	Organophosphorus	1.10	1.02	1.19	4.85 × 10^−02^
1,3-Dichloropropene	Halogenated organic	1.11	1.02	1.19	4.90 × 10^−02^

Fixed effect meta-analysis: adjusted for age at diagnosis/interview, gender, ethnicity (non-Hispanic White, Hispanic, Other), education (in years), smoking status (former smoker, current smoker, and non-smoker), study wave (PEG1 & PEG2), and index year.

In the sensitivity analysis using the weighted duration measure (i.e. $\frac{{{N_i}}}{{{T_i}}} \times {\mathrm{log}}\left( {{N_i} + 1} \right)$), all 56 pesticides initially identified remained positively associated with PD at FDR < 0.05, and the same pesticides had the largest estimated effect sizes (i.e., endothall, pyriproxyfen, phorate, sodium chlorate, and dicofol). Moreover, this alternative exposure metric found 10 additional pesticides to be associated with increased PD risk at FDR < 0.05, including copper hydroxide, disulfoton, glyphosate (isopropylamine salt), 4-(2,4-Db) (dimethylamine salt), endosulfan, fluazifop-butyl, oxyfluorfen, bacillus thuringiensis (berliner), azinphos-methyl, and simazine (supplemental table 2).

For the analyses excluding participants with short exposure histories, results remained consistent with the primary analyses. Under the 10 year threshold scenario, four chemicals were no longer identified, including 2,4-D (dimethylamine salt), dinoseb, dicamba (dimethylamine salt) and 1,3-dichloropropene. Under the 5 year threshold scenario, six chemicals (2,4-D (dimethylamine salt), mineral oil, dinoseb, dicamba (dimethylamine salt), diazinon, 1,3-dichloropropene) were no longer identified (supplemental table 2).

### 3.3. Comparison between duration and intensity results

**Figure 1.**
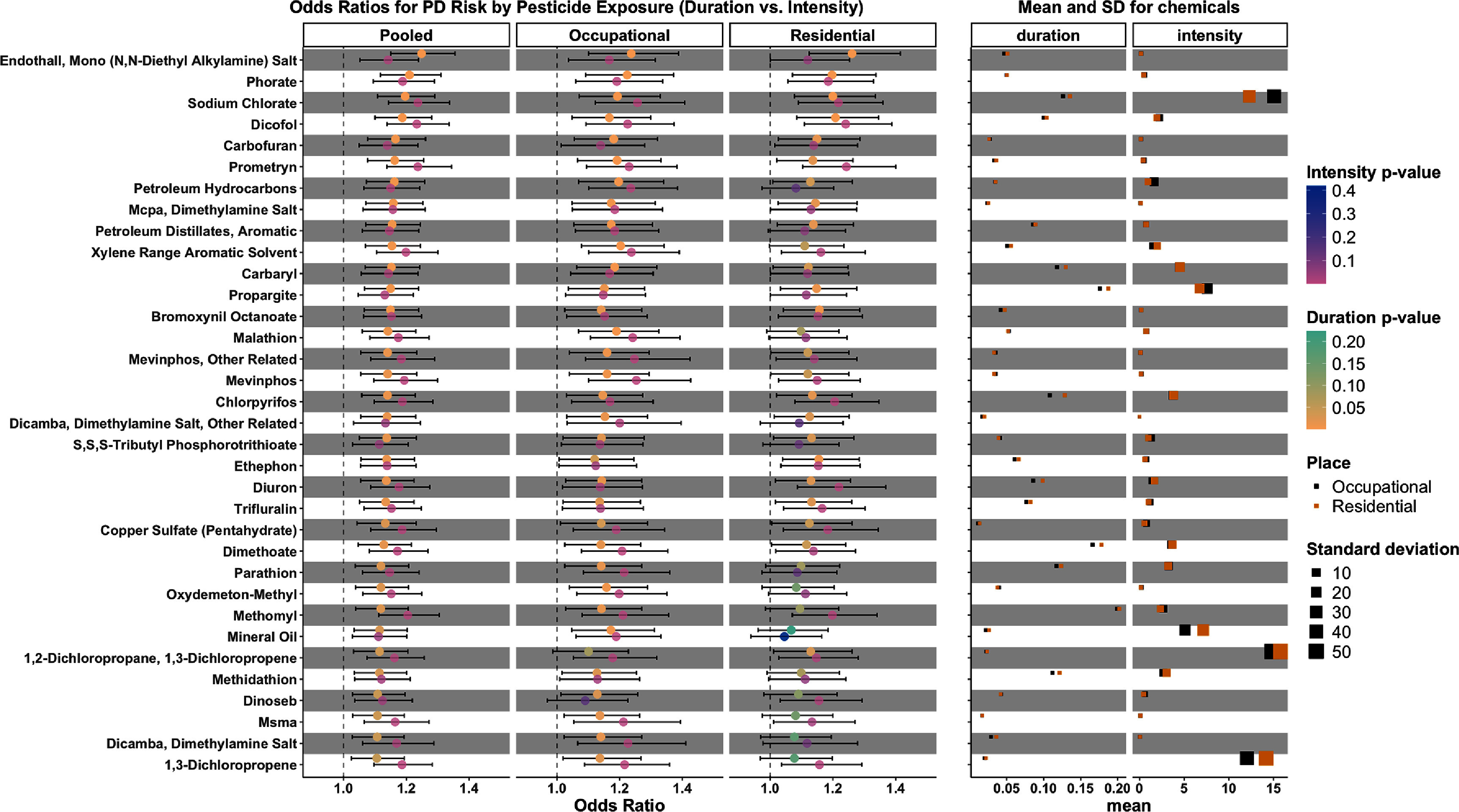
Overview of the fixed-effect meta-analysis (‘Pooled’ column in figure) and unconditional logistic regression results separate for exposure assessed at workplace (‘Occupational’ column) and residence (‘Residential’ column). Pesticides were included if FDR < 0.05 using both duration and intensity measure (*n* = 34). Exposure: intensity/duration of pesticide exposure from 1974 to 10 years prior to the index year outcome: PD status (with PD vs without PD) Adjusted covariates: age, race/ethnicity, sex, education years, smoking status, index year (diagnosis year for patients; interview year for controls), study wave (PEG1 vs PEG2).

Comparing the 56 pesticides associated with PD based on duration (FDR < 0.05) and the 53 PD-related pesticides identified using application intensity [9], 22 were associated only with duration and 19 were associated only with intensity. The remaining 34 pesticides are presented in figure 1, showing ORs, confidence intervals, means, and SD for both exposure metrics. The meta-analysis results indicated that higher pesticide exposure, whether assessed by duration or intensity, was associated with increased PD risk (ORs 1.0–1.4). Stratified analyses for occupational and residential exposures yielded similar trends, with some variation between settings. The effect estimates for both metrics of duration/intensity-only associated pesticides are shown in supplemental figures 1 and 2.

**Figure 2.**
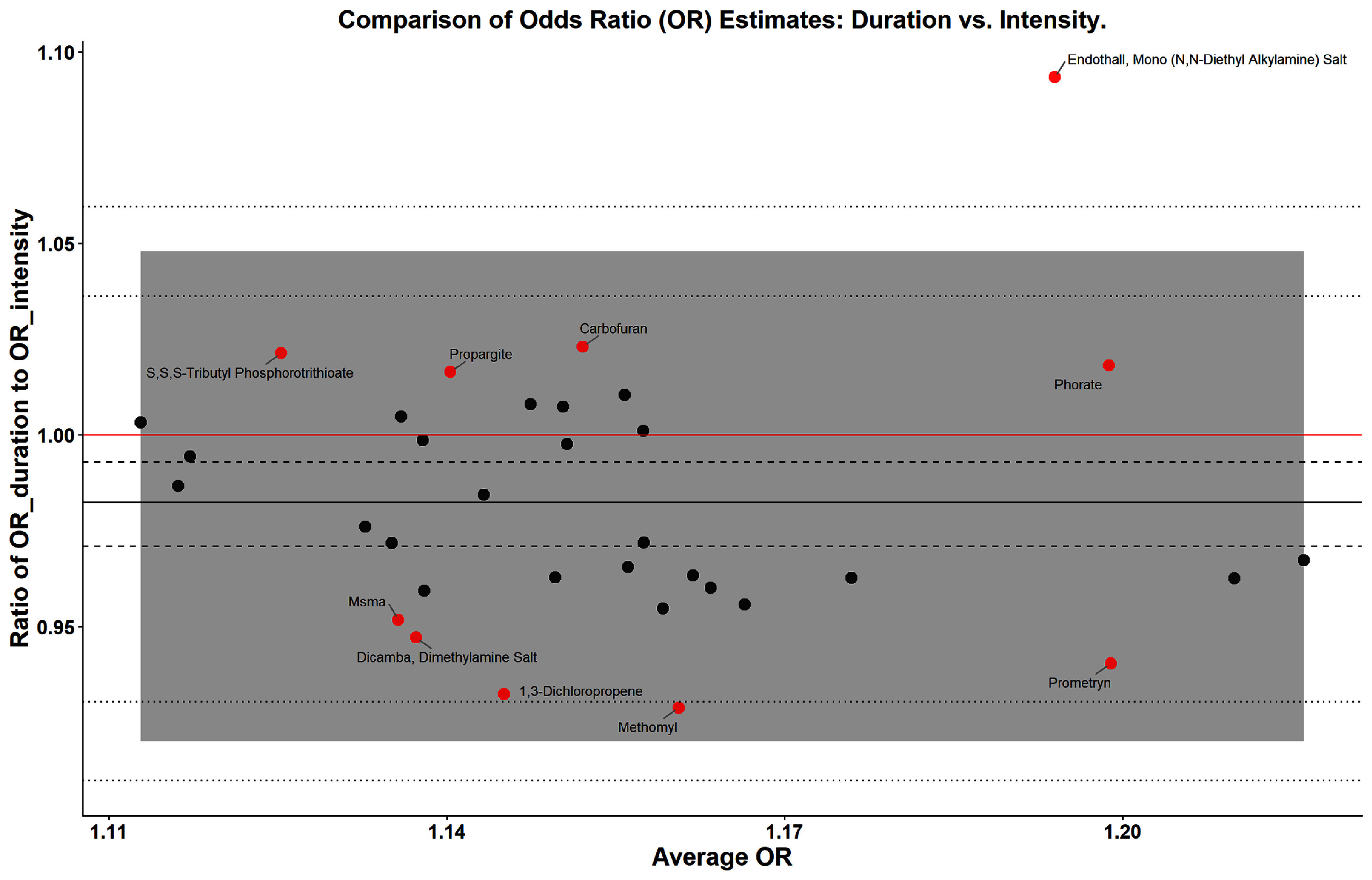
Comparison of odds ratio (OR) estimates: duration vs intensity. The Bland–Altman plot illustrates how the ratio of ORs from duration-based and intensity-based measurements varies with the average OR, calculated as the geometric mean of the two estimates. The red horizontal line indicates no difference (i.e., a ratio of 1). The shaded area represents the 95% Bland–Altman limits of agreement, showing the expected range of variation between the two measurement methods. The dashed lines denote the statistical precision around the average ratio (bias), while the dotted lines mark the precision around the limits of agreement. Red points highlight pesticides with the largest deviations from equivalence between duration- and intensity-based OR estimates and are labeled for clarity.

Figure 2 compared the OR estimates of the 34 pesticides identified from duration- and intensity-based pesticide exposure metrics using a Bland–Altman plot. The plot showed that most data points were within the 95% limits of agreement (shaded area), suggesting good overall agreement between the two metrics. The average ratio of ORs is close to 1.0, as indicated by the black solid horizontal line. However, the paired *t*-test suggested that the estimated effect sizes for duration were on average slightly smaller than those for intensity (${\mathrm{O}}{{\mathrm{R}}_{{\mathrm{duration}}}}$ to ${\mathrm{O}}{{\mathrm{R}}_{{\mathrm{intensity}}}}$ ratio = 0.98, 95% CI: 0.97–0.99, paired *t*-test *p* = 0.003). A broader comparison of all 75 pesticides (the union of the 56 duration-related and 53 intensity-related pesticides) likewise demonstrated strong agreement between the two metrics, with duration estimates again slightly smaller on average (supplemental figure 3). Sensitivity analysis using the weighted duration measure produced similarly high agreement with the intensity metric (supplemental figure 4).

Additionally, a positive correlation was observed between duration- (both unweighted and weighted) and intensity-based exposure metrics for these 75 pesticides, with a Pearson correlation coefficient of 0.28 (*p* = 0.015) and 0.29 (*p* = 0.012), respectively (supplemental figure 5).

### 3.4. The lagged effect of pesticides on PD in constrained DLMs

**Figure 3.**
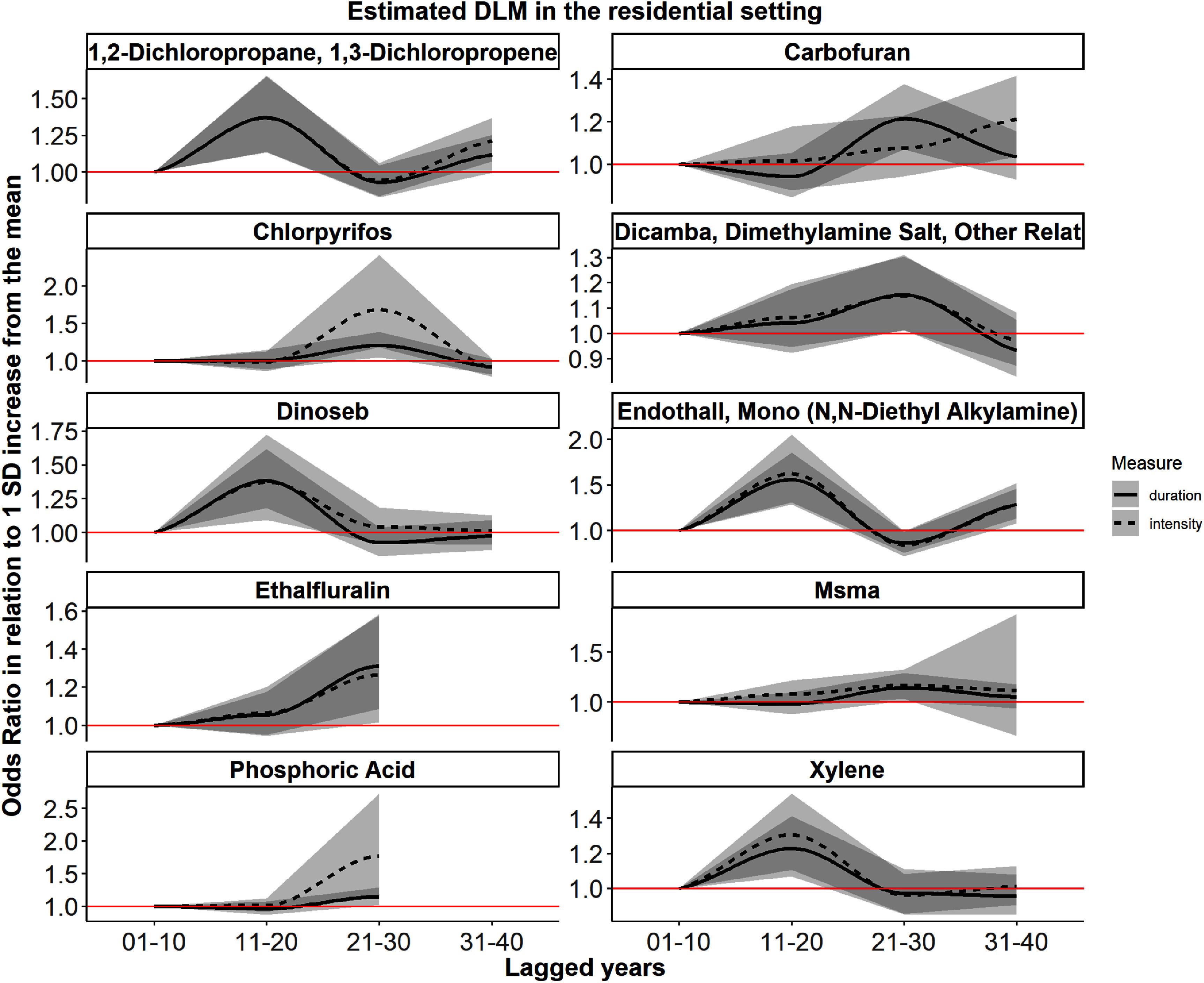
Estimated DLM with spline functions for chemicals with at least 1 period that has a CI does not include 1 in the residential setting. The *y*-axis shows the odds ratio in relation to 1 SD increase from the mean level in pesticide exposure; the *x*-axis depicts lagged-year intervals. The black solid line shows the predicted OR using duration measurement, the black dashed line shows the predicted OR using intensity measurement, the grey area indicates the 95% confidence interval, the red line indicated the reference OR = 1. Exposure: duration/intensity of pesticide exposure in each 10 year lagging interval outcome: PD status (with PD vs without PD) adjusted covariates: age, race/ethnicity, sex, education years, smoking status, index year (diagnosis year for patients; interview year for controls), study wave (PEG1 vs PEG2).

When applying DLM analysis with spline functions, we identified 10 pesticides (1,2-dichloropropane, carbofuran, chlorpyrifos, dicamba, dinoseb, endothall, ethalfluralin, msma, phosphoric acid, and xylene) out of the above 56 PD-related pesticides using both duration and intensity measures for which the estimated effect in at least 1 exposure window was stronger and formally statistically significant while adjusting for the same covariates as in the logistic regression model. Among the remaining 46 pesticides, 7 could not be estimated due to their short exposure history (i.e., with only 1 exposure window after applying a lag period), 18 had lagged effects with duration only, while 4 showed lagged effects with intensity only. The remaining 17 pesticides also exhibited stronger OR estimates in at least one lagged window, although the effects did not reach formal statistical significance. Model estimates are shown in supplemental table 2. Figure 3 shows the trends of OR changes across time-windows for these 10 pesticides. In general, for most pesticides the duration or intensity-based measures showed a similar pattern of changing ORs over time and had the same lag period, except for carbofuran. Chlorpyrifos, dicamba, ethalfluralin, msma, and phosphoric acid exhibited the highest odds for PD in the 21–30 years exposure window prior to diagnosis, while for 1,2-dichloropropane, dinoseb, endothall, and xylene, the time-window closer to diagnosis showed the strongest associations (i.e. 11–20 years window). For endothall, there were two peaks (i.e. 11–20 and 31–40 years) associated with increasing PD risk, where the first peak was slightly stronger than the second.

Sensitivity analyses using different modeling strategies suggested that our results are consistent across models and the constrained DLM model with spline functions. The results for these additional models are shown in supplementary figure 6.

## 4. Discussion

Our findings reveal novel associations between long-term chronic pesticide exposure and PD, with 56 of 287 examined pesticides showing positive associations in a duration-based analysis (FDR < 0.05) in one metric and an additional 10 pesticides were associated with our alternate weighted duration metric. Among these, OPs and petroleum derivatives were most frequently implicated. Notably, for endothall, a protein phosphatase 2a inhibitor, we estimated the largest effect size (OR = 1.25 per SD, 95% CI = 1.15–1.35). While there was strong overall agreement between duration- and intensity-based measures, as evidenced by the Bland–Altman plot (figure 2; supplemental figure 3), effect sizes for duration were slightly smaller than those for intensity on average. Furthermore, a latency period of either 11–20 years or 21–30 years was identified using both duration and intensity measures.

This study builds on our previous work, in which we identified 53 pesticides associated with PD at FDR < 0.05 employing an untargeted design with an exposure intensity measure that represented the average pounds of active ingredient applied per year. The previous study further characterized co-exposure patterns and demonstrated direct dopaminergic neurotoxicity for 10 lead pesticides [9]. In the current study, we compared the OR estimates of PD-related pesticides (*n* = 34) identified by both intensity and duration of exposure. Notably, we observed similar OR estimates no matter whether we used intensity (average annual pounds applied) or duration (the proportion of years with any exposure to an agent in the exposure window) measure, suggesting that both intensity and duration exposure measurements are likely important for these pesticides related to PD pathogenesis, and that long-term exposures over decades are of concern.

Our top hit for the duration of exposure metric was endothall. Endothall has been registered for use in California since at least the 1960s. In 1984, approximately 87 000 pounds of this formulation were applied in the state, primarily for the defoliation of cotton [27–29]. Endothall also helps manage weeds and is employed in water management [27]. Currently, this agent is no longer registered in California according to the California Department of Pesticide Regulation (CADPR) [30]. While no other epidemiologic studies associated endothall with PD, our previous experimental work showed that endothall is directly toxic to midbrain dopaminergic neurons from patient-derived induced pluripotent stem cells (iPSCs) and that this toxicity was somewhat specific to neurons, as endothall was not toxic to cardiomyocytes derived from the same iPSC lines [9].

In addition, we identified 14 OP pesticides that were positively associated with PD in our primary analysis. This is not surprising since OPs not only inhibit acetylcholinesterase (AChE) but also induce oxidative stress and mitochondrial dysfunction, leading to excessive accumulation of acetylcholine and eventually neurodegeneration [31–33]. In addition, more recent evidence suggests that OPs, such as chlorpyrifos, may induce neuronal loss by impairing autophagy [34]. Interestingly, petroleum derivatives were also highlighted as risk factors for PD in residential and occupational ambient exposure settings. These petroleum-based products are often used as industrial solvents [35], and in agricultural settings, they have been used as pesticide solvents or as adjuvants to enhance the effect of active ingredients by, for example, acting as surfactants [36]. Although they are not banned outright, they are not actively registered according to CADPR [30]. In support of our results, a meta-analysis of 13 case-control studies that included 3020 PD patients and 6494 controls reported a summary OR of 1.32 (95% CI 1.08–1.62) for occupational hydrocarbon exposure [37].

The DLM models suggest there are potential windows when exposure is most relevant, and that this may differ by pesticides such that for some the period of 11–20 years and for others the period 21–30 years prior to diagnosis are seemingly more important for the development of PD. In sensitivity analyses, we found results to be roughly consistent with the constrained DLM when using spline functions and different regression models indicated that lags are relatively robustly estimated. Although collinearity is still a possibility for DLMs in general [38], our DLM estimates were similar to those derived from ridge regression models (designed to handle multicollinearity) in most scenarios, suggesting that collinearity of pesticide exposure across time periods was limited in this analysis.

A major advantage of this study was that our exposure assessment did not rely on participant recall. Instead, we made use of commercial pesticide application records from the CA-PUR and a GIS model, which enabled us to assess historic ambient exposure for individual chemicals with a record-based assessment, thus minimizing potential recall bias. In addition, duration of exposure captures the persistence of nearby applications and is less sensitive to fluctuations in the quantity of pesticide applied per acre, which may vary due to factors like weather, crop type, or application method, etc. Additionally, the DLM method helps decipher which periods of exposure are most related to PD, supporting the notion that chronic pesticide exposures contribute to disease pathogenesis with differing latency periods. Furthermore, each patient in our study was examined by UCLA neurologists/movement disorder specialists to confirm the PD diagnosis, which limited outcome misclassification.

However, there are also some limitations of this study. First, our proximity-based metrics are surrogates for potential ambient exposure pathways and cannot capture individual behaviors, microenvironments, or absorbed dose. Second, the exposure assessment was constrained by the length and completeness of residential and occupational address histories available since 1974 and the variability of enrollment timing, resulting in heterogeneous observable exposure windows. Thus, our primary duration metric is a proportion of eligible years, selected to standardize across participants with different observable exposure windows. As a result, short and long windows with the same proportion can receive similar values. We therefore provided a weighted-duration sensitivity analysis that incorporates the number of exposed years. Then, we also excluded individuals with limited exposure windows to assess the robustness of the primary findings to limited observable exposure periods. Lastly, correlations between pesticide exposures are present in the current study, as some pesticides were applied in the same season on the same fields or at different times but still in the same proximity to study participants’ homes or workplaces. This opens the possibility of associations being confounded by other pesticide exposures. For example, the associations that we observed could be partially attributable to a co-applied pesticide with PD-related neurotoxicity or potentially co-exposure to several pesticides may be most relevant. Therefore, chemical mixtures studies are needed in future research to explore the synergistic effect of co-applied pesticides and the potential mechanism regarding their neurotoxicity.

In conclusion, this study demonstrates that the duration of long-term ambient pesticide exposure, independent of application intensity, is an important contributor to PD risk. By focusing on the proportion of years with nearby pesticide application, our findings underscore the etiologic relevance of persistent, chronic exposure over decades rather than short-term or episodic exposure alone. The identification of stronger associations for exposures occurring two to three decades prior to diagnosis supports the presence of periods of heightened susceptibility in PD pathogenesis, consistent with the disease’s long preclinical course. Together, these findings strengthen the evidence linking agricultural pesticide exposure to PD and highlight the importance of incorporating both pesticide exposure persistence and timing into future PD research.

## Data Availability

The data cannot be made publicly available upon publication because they contain sensitive personal information. The data that support the findings of this study are available upon reasonable request from the authors. Supplementary table 1 available at http://doi.org/10.1088/2752-5309/ae4645/data1. Supplementary table 2 available at http://doi.org/10.1088/2752-5309/ae4645/data2. Supplementary figures available at http://doi.org/10.1088/2752-5309/ae4645/data3. PEG study recruitment available at http://doi.org/10.1088/2752-5309/ae4645/data4.
